# Circular RNA circVAPA contributes to non-small-cell lung cancer progression via miR-342-3p-dependent regulation of ZEB2

**DOI:** 10.1186/s12957-021-02447-4

**Published:** 2021-11-29

**Authors:** Xiaoyang Liu, Yang Cheng, Yan Wang, Yinhong Zhang

**Affiliations:** Department of Respiratory and Critical Care Medicine, Beijing Jishuitan Hospital, No. 31 Xinjiekoudong Street, Xicheng District, Beijing, 100035 China

**Keywords:** NSCLC, circVAPA, miR-342-3p, ZEB2

## Abstract

**Background:**

Accumulating evidence demonstrated that circular RNAs (circRNAs) play pivotal regulatory roles in the pathology of cancers. Disclosing the roles and molecular mechanisms of circRNAs in tumorigenesis and development is essential to identify novel diagnostic and therapeutic targets. In this study, we explored the role of circVAPA in non-small-cell lung cancer (NSCLC) progression and its associated mechanism.

**Methods:**

The expression level of RNA was analyzed by real-time quantitative polymerase chain reaction (RT-qPCR). Cell proliferation was assessed by MTT assay and colony-forming assay. Cell apoptosis was analyzed by flow cytometry. Cell migration and invasion were assessed by transwell assays. Dual-luciferase reporter, RNA pull-down, and RNA immunoprecipitation (RIP) assays were used to test the intermolecular interactions. The role of circVAPA was assessed in vivo. And xenograft tumor tissues were analyzed by immunohistochemistry (IHC) staining.

**Results:**

CircVAPA expression was upregulated in NSCLC tissues and cell lines, and a high level of circVAPA was associated with a poor prognosis of NSCLC patients. CircVAPA silencing suppressed the proliferation, migration, and invasion and induced the apoptosis of NSCLC cells. CircVAPA served as a molecular sponge for microRNA-342-3p (miR-342-3p). miR-342-3p interference largely reversed circVAPA knockdown-mediated anti-tumor effects in NSCLC cells. Zinc finger E-box-binding homeobox 2 (ZEB2) was a target of miR-342-3p, and miR-342-3p overexpression suppressed the malignant behaviors of NSCLC cells largely by downregulating ZEB2. CircVAPA silence repressed xenograft tumor growth in vivo, and IHC assay confirmed that circVAPA silence restrained the proliferation and metastasis but induced the apoptosis of NSCLC cells in vivo.

**Conclusion:**

CircVAPA contributes to the progression of NSCLC by binding to miR-342-3p to upregulate ZEB2. CircVAPA/miR-342-3p/ZEB2 axis might be a novel potential target for NSCLC treatment.

## Background

Non-small-cell lung cancer (NSCLC) is the most common histological type of lung cancer, accounting for 80% of all lung cases [1]. Although considerable advancement has been made on the diagnosis and treatment of NSCLC, the clinical outcome of NSCLC patients is still unsatisfactory [2]. The development of novel diagnostic and prognostic targets is under active investigation for NSCLC [3]. Therefore, disclosing the pathogenesis of NSCLC is essential to identify novel diagnostic and therapeutic targets.

Circular RNAs (circRNAs) are a class of endogenous RNA molecules with a closed circular structure [4]. In recent years, circRNAs have shown pivotal regulatory roles in the initiation and development of NSCLC [5]. For instance, circ_0016760 is reported to be a novel indicator for the prognosis of NSCLC patients, and the upregulation of circ_0016760 significantly promotes cell growth and motility in NSCLC cells [6]. In this study, we focused on the role of circVAPA (hsa_circ_0006990) in NSCLC progression and its associated mechanism. Previous studies have reported the oncogenic role of circVAPA in multiple cancers, including colorectal cancer [7, 8], hepatocellular carcinoma [9], and breast cancer [10]. Nevertheless, the biological function of circVAPA in NSCLC remains unknown.

MicroRNAs (miRNAs), a group of small single-stranded RNA molecules, can regulate gene expression by base-pairing with their target messenger RNAs (mRNAs) [11]. MiRNAs are found to be widely dysregulated in various cancers, implying the important regulator roles of miRNAs in tumorigenesis and progression [12, 13]. Several miRNAs that might be potential biomarkers for NSCLC have been identified in NSCLC [14, 15]. For instance, miR-216b expression was closely related to ^18^F-FDG uptake and could be used for the classification and staging of NSCLC [16]. MiR-194-5p could enhance the sensitivity of NSCLC cells to DOX by regulating hypoxia-inducible factor-1 [17]. Through bioinformatics analysis, we found that miR-342-3p was a possible target of circVAPA in this study. The diagnosis value and prognostic significance of miR-342-3p have been unveiled in multiple malignant tumors [18, 19]. Qin *et al*. demonstrated that the expression of miR-342-3p is significantly reduced in the serum samples of NSCLC patients compared with controls, and serum miR-342-3p might be a novel promising biomarker for NSCLC [20]. Here, we explored the target relationship between circVAPA and miR-342-3p and further explored their functional correlation in NSCLC progression.

Through bioinformatics analysis, zinc finger E-box-binding homeobox 2 (ZEB2) was predicted as a candidate target of miR-342-3p. ZEB2 is a member of the ZEB family, and it plays an important role in regulating cell mobility [21]. Not surprisingly, accumulating evidence demonstrated that ZEB2 behaves as an oncogene through promoting the metastasis of lung cancer cells [22, 23]. In this study, we first analyzed the expression pattern of circVAPA in NSCLC tissues and cell lines. Loss-of-function experiments were conducted to analyze the functions of circVAPA in NSCLC cells. Then, we hypothesized that circVAPA played a regulatory role in NSCLC progression by regulating the miRNA/mRNA axis. The working mechanism of circVAPA was verified by rescue experiments. Therefore, the research was recognized to determine the regulatory relationships among circVAPA, miR-342-3p, and ZEB2 in NSCLC.

## Materials and methods

### Clinical samples

NSCLC tissue specimens and paired noncancerous tissue specimens were harvested from 66 NSCLC patients who had undergone surgical resection at Beijing Jishuitan Hospital. The serum samples were collected from 30 healthy volunteers and 35 NSCLC patients. Tissues and serum samples were stored at −80 °C until use. The written informed consents had been signed by all the participants before surgical resection and blood collection, and this study was authorized by the Ethics Committee of Beijing Jishuitan Hospital and carried out according to the guidelines of the Declaration of Helsinki. To analyze the correlation between the expression of circVAPA and the survival of NSCLC patients, 66 NSCLC patients were assigned to two groups with the median value of circVAPA expression as the cutoff.

### Cell lines

NSCLC cell lines (Calu-3, Calu-6, A549, and H1299) and normal human bronchial epithelial cell lines (HBE1) were purchased from Nanjing KeyGen Biotech (China). All cell lines were cultured with RPMI 1640 medium (Wisent, Shanghai, China) supplemented with 10% fetal bovine serum (FBS; Biochrom KG, Berlin, Germany) at 37 °C with 5% CO_2_.

### Real-time quantitative polymerase chain reaction

RNA isolation kit (Vazyme, Nanjing, China) was used to isolate RNA samples. The quality of RNA samples was assessed using the UV-3100PC spectrophotometer. cDNA was synthesized using RNA Reverse Transcription kits (Thermo Fisher Scientific, Carlsbad, CA, USA). RT-qPCR detection reagents (ABI, Foster City, CA, USA) were used for RT-qPCR analysis under a 7500 Real-Time PCR System (ABI). The expression of circRNAs and mRNAs was normalized to GAPDH, while the expression of miRNAs was normalized to U6. The relative fold changes were calculated by the 2^−ΔΔCt^ method. The sense and anti-sense sequences of primers are shown as below:

circVAPA (5′-GGAAGCTGTGTGGAAAGAGG-3′; 5′-GGCGAGGTGCTGTAGTCTTC-3′);

VAPA (5′-AGCTGTGTGGAAAGAGGCAA-3′; 5′-GCAGGTGTTGCAACTGTGTT-3′);

miR-342-3p (5′-GCCGAGTCTCACACAGAAAT-3′; 5′-CTCAACTGGTGTCGTGGA-3′);

ZEB2 (5′-CCTCTGTAGATGGTCCAGTGA-3′; 5′-GTCACTGCGCTGAAGGTACT-3′);

GAPDH (5′-CCTGTTCGACAGTCAGCCG-3′; 5′-GAGAACAGTGAGCGCCTAGT-3′);

U6 (5′-GCTTCGGCAGCACATATACTAAAAT-3′; 5′-CGCTTCACGAATTTGCGTGTCAT-3′).

### Cell transfection

The short hairpin RNA targeting circVAPA (sh-circVAPA#1, 5′-ATAAATTGGCCCCTTCACAGA-3′, sh-circVAPA#2, 5′-AATGATAAATTGGCCCCTTCA-3′, and sh-circVAPA#3, 5′-AATTGGCCCCTTCACAGATGT-3′), ZEB2-overexpression vector (ZEB2), miR-342-3p mimics (miR-342-3p, 5′-UCUCACACAGAAAUCGCACCCGU-3′), miR-342-3p inhibitors (anti-miR-342-3p, 5′-ACGGGUGCGAUUUCUGUGUGAGA-3′), and respective controls (sh-NC, vector, miR-NC, and anti-NC) were purchased from Sangon (Shanghai, China). NSCLC cells were seeded onto 24-well plates at the density of 5 × 10^3^ cells/well, and transient transfection was conducted with Lipofectamine 2000 (Sigma, San Francisco, CA, USA). The transfection efficiency was assessed at 48 h post-transfection.

### Cell proliferation assay

3-(4,5-Dimethyl-thiazol-2-yl)-2,5-diphenyltetrazolium bromide (MTT) assay was performed to analyze cell proliferation ability. NSCLC cells were seeded onto 96-well plates (4000 cells/per well). Subsequently, 20 μL of MTT (Sigma) was added to cells. The formazan crystals were dissolved in dimethyl sulfoxide solution. The absorbance at the wavelength of 490 nm was examined under the microplate reader (Bio-Rad, Hercules, CA, USA).

For the colony-forming assay, NSCLC cells were seeded onto 6-well plates (600 cells/ well) for 14 days, followed by staining with 0.5% crystal violet solution (Sigma). Then, the number of colonies was manually counted under an inverted microscope (Olympus, Tokyo, Japan).

### Flow cytometry assay

Transfected A549 and H1299 cells were harvested and cell apoptosis was analyzed using Annexin V-FITC Apoptosis Detection Kit (Thermo Fisher Scientific) referring to the producer’s instructions. NSCLC cells were simultaneously stained with Annexin V-FITC and PI for 15 min in the dark. Cell samples were loaded onto a FACS Calibur flow cytometer (ABI), and the apoptosis rate was then analyzed.

### Transwell assay

For transwell assay, 600 μL of medium with 10% FBS and 200 μL of serum-free media containing 5 × 10^3^ cells were respectively introduced into the lower and top chambers of 24-well transwell chambers. Following incubation at 37 °C for 24 h, the invaded cells in the chambers were stained and then counted under the microscope (Olympus; 100× amplification). As for invasion assay, a 24-well transwell chamber was additionally adhered with Matrigel (BD Biosciences, Franklin Lakes, New Jersey, USA).

### Western blot assay

Briefly, the total proteins were isolated by RIPA buffer (Thermo Fisher Scientific). After centrifuging for 10 min (12000 g, 4 °C), SurePAGE gels were carried out to isolate total proteins, followed by a wet-transfer process. The membranes (Millipore, Billerica, MA, USA) were blockaded by 3% Albumin Bovine V and then interacted with specific antibodies purchased from CST (Danvers, MA, USA): E-cadherin (#3195S; 1:1500), N-cadherin (#13116S; 1:1500), Vimentin (#5741S; 1:1500), GAPDH (#2118S; 1:1500), and ZEB2 (#3396S; 1:1500). After incubation for 24 h at 4 °C, membranes were incubated with secondary antibodies (#7074S; 1:2000; CST). Finally, an ECL Substrate Kit (Bio-Rad) was used to visualize western bands.

### Dual-luciferase reporter assay

The complementary sequences between circVAPA and miR-342-3p were displayed by circBANK (http://www.circbank.cn/) and Starbase (http://starbase.sysu.edu.cn/). The binding regions of miR-342-3p within ZEB2 were predicted by Starbase. The partial sequences of circVAPA or ZEB2 3′UTR containing miR-342-3p interacting sites were amplified and inserted into the pGL3-basic vector (EK-Bioscience, Shanghai, China). NSCLC cells were seeded into 24-well plates at 5 × 10^3^ cells/well and transfected with a vector according to the experimental design by Lipofectamine 2000 (Sigma). NSCLC cells were harvested at 48 h post-transfection for luciferase activity detection.

### RNA pull-down and RNA immunoprecipitation assay

The 50 nM of biotin-labeled miR-342-3p (Bio-miR-342-3p; GenePharma, Shanghai, China) were transfected into 1 × 10^6^ NSCLC cells for 48 h before harvest, with Bio-miR-NC as the negative group. After incubated with lysis buffer, 100 μL of cell lysates was pulled down streptavidin magnetic beads (Life Technologies, Darmstadt, Germany) for 3 h. After centrifugation at 10000 g for 15 min, the biotin-coupled RNA complex was subjected to RT-qPCR analysis. In addition, Magna RIP Kit (Millipore) was performed in RIP assay. In brief, cell lysates went through a 24-h incubation with magnetic beads embracing Ago2 or IgG at 4 °C for 24 h. After digesting with proteinase K, the target gene was confirmed by RT-qPCR analysis.

### In vivo experiment

Four weeks of BALB/c nude mice (Vital River Laboratory, Beijing, China) were injected subcutaneously with A549 cells stably transfected with sh-circVAPA#1 (5×10^6^/100 μL of medium/mice), with sh-NC as control, respectively named as sh-circVAPA#1 group (*n* = 6) and sh-NC group (*n* = 6). Tumor volume was monitored using calipers based on volume = 1/2 (length × width^2^). All animal studies were performed with authorization by the Institutional Animal Care and Use Committee of Beijing Jishuitan Hospital and performed in accordance with the guidelines of the National Animal Care and Ethics Institution.

### Statistical analysis

All experiments were independently conducted at least 3 times and all data were displayed as mean ± standard deviation. *P*-value was calculated using Student’s *t*-test (in two groups) or one-way analysis of variance with Tukey post hoc test (in multiple groups), and *P* less than 0.05 was considered the threshold of significance. Receiver operating characteristic (ROC) curve analysis was used to assess the diagnostic value of circVAPA. Pearson’s correlation analysis was used to analyze the linear correlation among circVAPA, miR-342-3p, and ZEB2.

## Results

### CircVAPA is abnormally upregulated in NSCLC tissues and cell lines

**Fig. 1:**
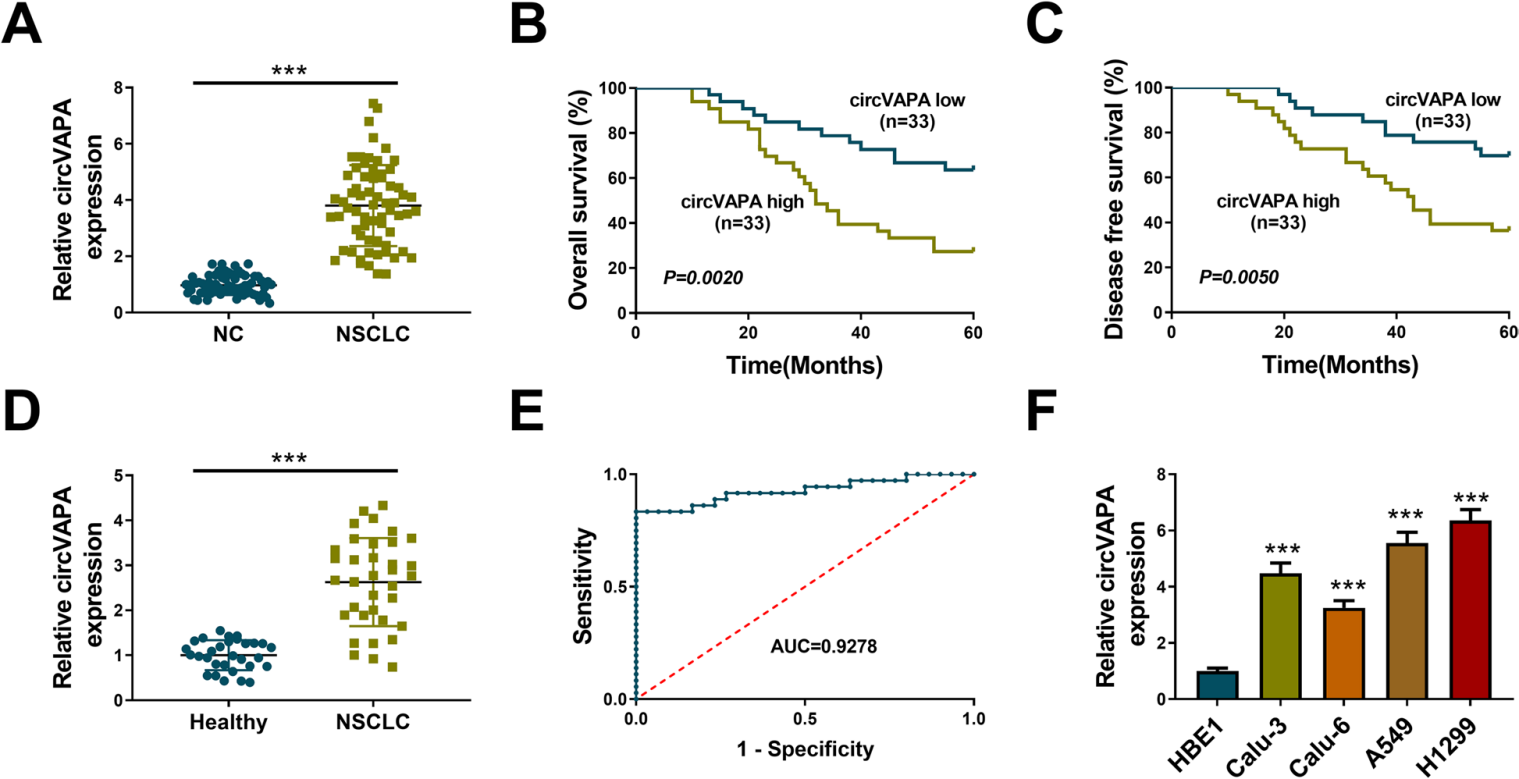
CircVAPA is abnormally up-regulated in NSCLC tissues and cell lines. **A** The relative expression level of circVAPA was measured by RT-qPCR assay in NSCLC tissues and neighboring normal tissues. **B**, **C** The survival curves of NSCLC patients were shown. **D** The RT-qPCR assay was used to determine serum circVAPA in NSCLC patients and healthy volunteers. **E** The diagnostic value of circVAPA was assessed by Receiver Operating Characteristic (ROC) curve. **F** The expression level of circVAPA was detected by RT-qPCR assay in NSCLC cell lines and control. ***P < 0.001

To investigate the role of circVAPA in NSCLC progression, the expression pattern of circVAPA was first measured by RT-qPCR assay. The results suggested that circVAPA expression was notably increased in NSCLC tissues compared with adjacent normal tissues (Fig. 1A). To analyze the correlation between circVAPA level and the survival of NSCLC patients, NSCLC patients were divided into two groups with the mean value of circVAPA expression as the cutoff. We found that NSCLC patients with high circVAPA expression were associated with poor overall survival and disease-free survival (Fig. 1B, C), suggesting that circVAPA might be a novel prognostic indicator for NSCLC patients. The expression of circVAPA in serum samples was also upregulated in NSCLC patients compared with healthy volunteers (Fig. 1D). ROC curve data revealed that the area under the curve (AUC) reached 0.9278, indicating the diagnostic value of circVAPA in NSCLC (Fig. 1E). Furthermore, we found that circVAPA expression was upregulated in NSCLC cell lines relative to the HBE1 cell line (Fig. 1F), especially in A549 and H1299 cell lines. Therefore, A549 and H1299 cell lines were selected for the following experiments. These results suggested that circVAPA expression might be associated with NSCLC progression.

### Knockdown of circVAPA inhibits the proliferation and induces the apoptosis of NSCLC cells

**Fig. 2:**
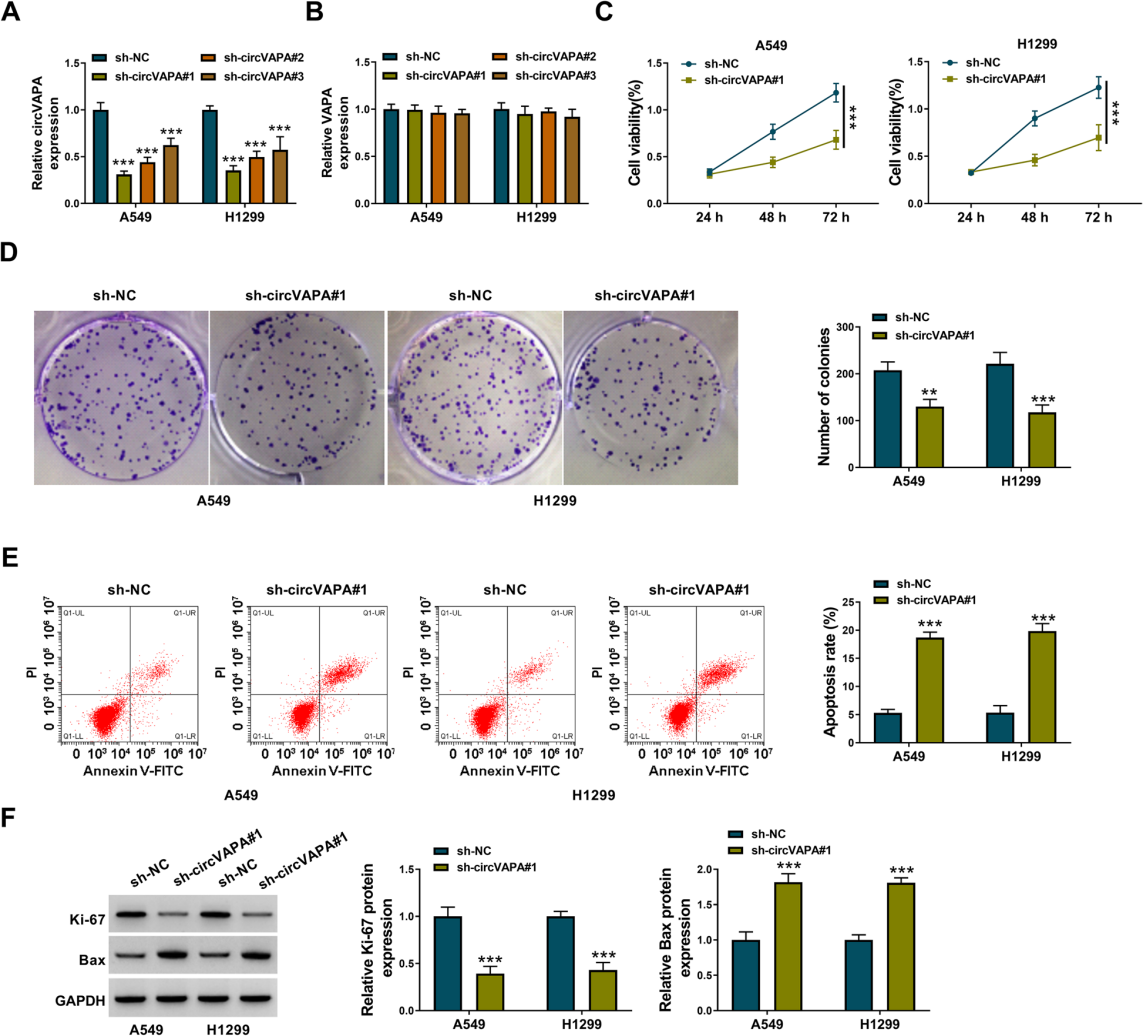
Knockdown of circVAPA inhibits the proliferation and induces the apoptosis of NSCLC cells. **A**, **B** The expression levels of circVAPA and VAPA were examined by RT-qPCR assay in A549 and H1299 cells transfected with sh-NC, sh-circVAPA#1, sh-circVAPA#2, or sh-circVAPA#3. **C** The cell viability of A549 and H1299 cells was measured by MTT assay. **D** Colony-forming ability of A549 and H1299 cells was assessed by colony-forming assay. **E** The flow cytometry assay was performed to measure cell apoptosis. **F** Western blot assay was conducted to measure the expression of proliferation-associated protein (Ki-67) and pro-apoptotic protein (Bax). **P < 0.01, ***P < 0.001

A549 and H1299 cell lines stably transfected with sh-NC, sh-circVAPA#1, sh-circVAPA#2, or sh-circVAPA#3 were established to perform loss-of-function experiments. As presented in Fig. 2A, B, high transfection efficiencies and specificities of three circVAPA-specific shRNAs were confirmed in NSCLC cells. MTT assay revealed that circVAPA knockdown suppressed the proliferation of NSCLC cells (Fig. 2C). Similarly, the colony-forming assay showed that circVAPA interference markedly reduced the number of colonies (Fig. 2D), further demonstrating that circVAPA knockdown restrained the proliferation of NSCLC cells. Flow cytometry uncovered that the knockdown of circVAPA induced apoptosis of A549 and H1299 cells (Fig. 2E). The expression of the proliferation-associated protein (Ki-67) and pro-apoptotic protein (Bax) was determined by western blot assay. We found that circVAPA knockdown reduced the expression of Ki-67 while increasing the level of Bax (Fig. 2F), further demonstrating that circVAPA interference suppressed the proliferation and induced the apoptosis of NSCLC cells. Taken together, circVAPA facilitated the proliferation and suppressed the apoptosis of NSCLC cells.

### CircVAPA silencing restrains the migration and invasion of NSCLC cells

**Fig. 3:**
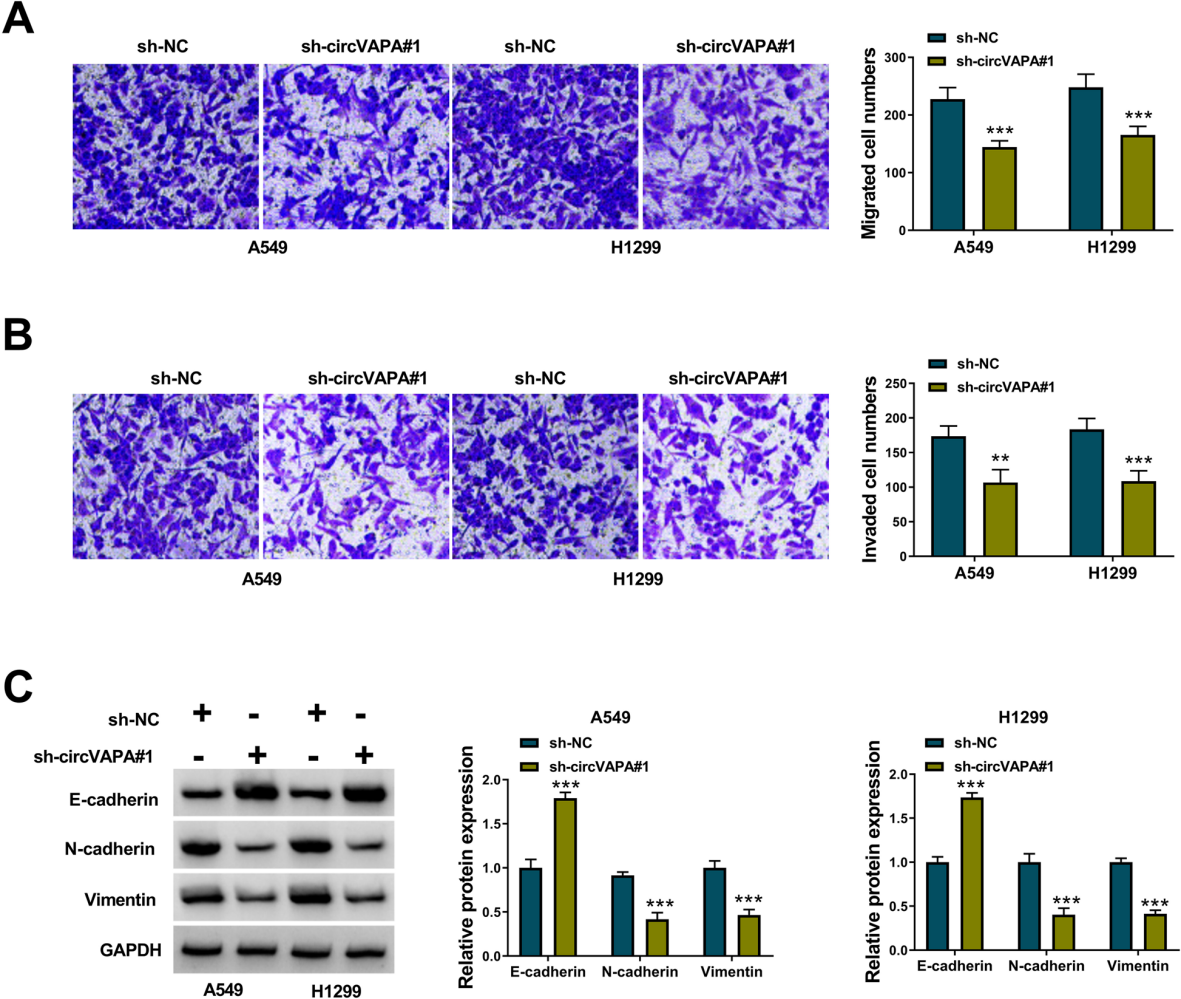
CircVAPA silencing restrains the migration and invasion of NSCLC cells. **A**, **B** The migration and invasion were determined by transwell assay in A549 and H1299 cells transfected with sh-NC, or sh-circVAPA#1. **C** The protein expression levels of E-cadherin, N-cadherin, and Vimentin were quantified by western blot analysis in A549 and H1299 cells transfected with sh-NC, or sh-circVAPA#1. **P < 0.01, ***P < 0.001

The migration and invasion abilities of tumor cells were highly associated with tumor invasiveness. We explored the role of circVAPA on the migration and invasion abilities of NSCLC cells. As presented in Fig. 3 A-B, circVAPA knockdown inhibited the migration and invasion abilities of A549 and H1299 cells, evidenced by the reduced numbers of migrated and invaded cells. Additionally, circVAPA interference increased E-cadherin expression while decreasing N-cadherin and Vimentin expression in A549 and H1299 cells (Fig. 3C). In summary, circVAPA silencing inhibited the migration and invasion of NSCLC cells.

### CircVAPA directly interacts with miR-342-3p

**Fig. 4:**
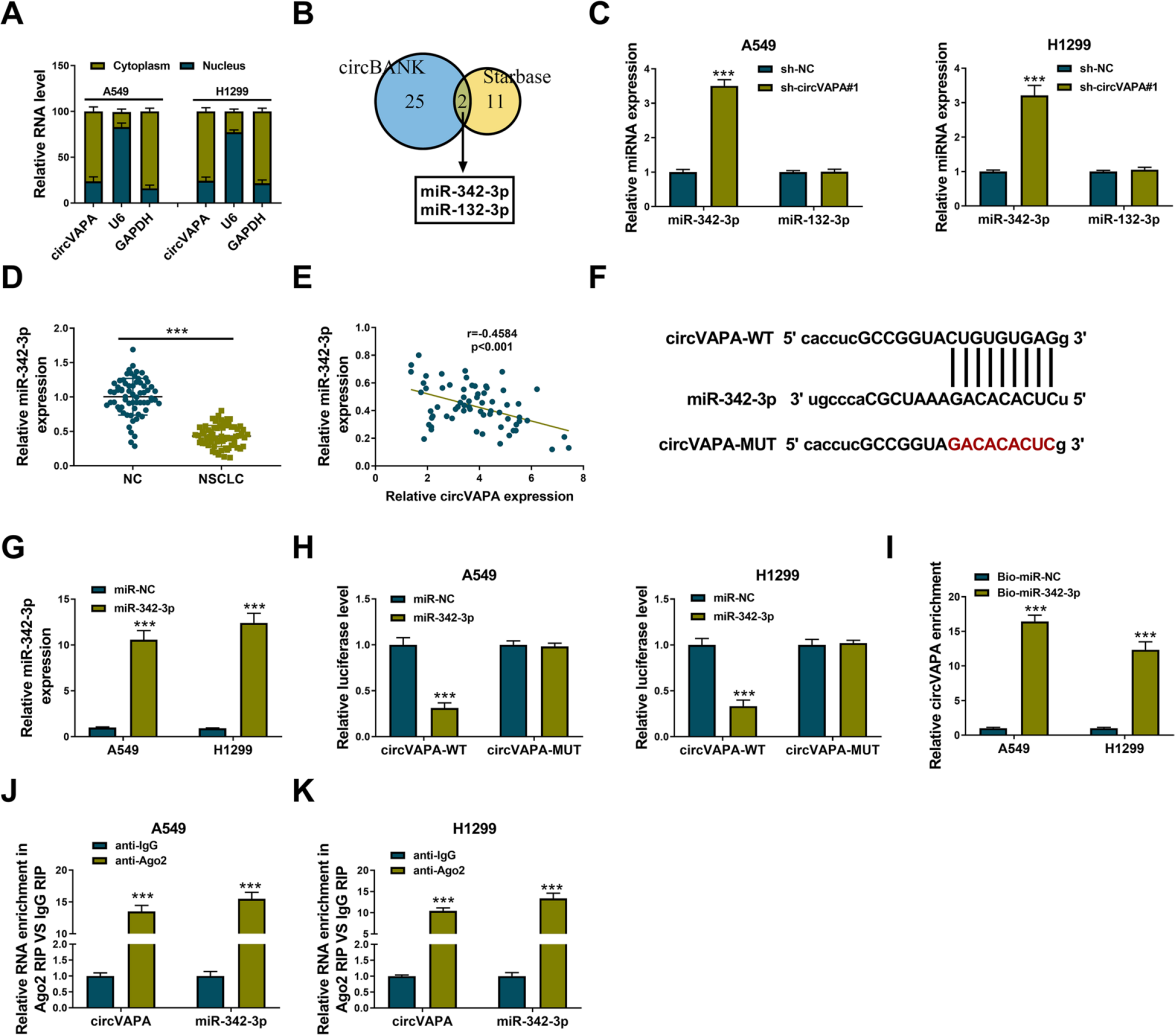
MiR-342-3p was a direct target of circVAPA. **A** Cytoplasmic and nuclear fraction RNAs were isolated, circVAPA expression was determined by RT-qPCR assay. **B** Schematic illustration showed the overlap of the target miRNAs of circVAPA predicted by circBANK and Starbase. **C** The expression levels of miR-342-3p and miR-132-3p were examined by RT-qPCR assay in A549 and H1299 cells transfected with sh-NC, or sh-circVAPA#1. **D** The expression of miR-342-3p was assessed by RT-qPCR assay in NSCLC tissues and neighboring normal tissues. **E** Pearson’s correlation analysis was used to analyze correlation between miR-342-3p and circVAPA. **F** Binding regions between miR-342-3p and circVAPA were shown. **G** The transfection efficiency of miR-342-3p was checked by RT-qPCR assay. **H** The relative luciferase activity was analyzed in A549 and H1299 cells. **I**–**K** The interaction association between miR-342-3p and circVAPA was confirmed by RNA pull-down and RIP assays. ***P < 0.001

CircVAPA was majorly localized in the cytoplasmic fraction of NSCLC cells (Fig. 4A), suggesting that circVAPA might serve as a miRNA sponge. Then, bioinformatics databases circBANK and Starbase were used to predict the possible miRNA targets of circVAPA. MiR-342-3p and miR-132-3p were predicted to be the targets of circVAPA by both databases (Fig. 4B). We found that circVAPA knockdown upregulated the expression of miR-342-3p in A549 and H1299 cells, while the expression of miR-132-3p was not affected by circVAPA silencing (Fig. 4C). Therefore, we selected miR-342-3p for further analysis. RT-qPCR data showed that miR-342-3p was downregulated in NSCLC tissues compared with adjacent normal tissues (Fig. 4D). The expression of miR-342-3p was negatively correlated with circVAPA expression in NSCLC tissues (Fig. 4E). The putative binding sequence between miR-342-3p and circVAPA predicted by circBANK and Starbase databases was displayed in Fig. 4F. The transfection efficiency of miR-342-3p mimics was significant in NSCLC cells (Fig. 4G). Furthermore, the overexpression of miR-342-3p reduced the luciferase activity of the wild-type reporter (circVAPA-WT) instead of the mutant reporter (circVAPA-MUT) (Fig. 4H), suggesting that circVAPA interacted with miR-342-3p via the predicted sites. RNA pull down assay suggested that circVAPA was pulled down when using Bio-miR-342-3p (Fig. 4I). RIP assay revealed that circVAPA and miR-342-3p were simultaneously enriched in the Ago2 antibody group compared with the IgG antibody group (Fig. 4J, K). The results of the dual-luciferase reporter assay, RNA pull down assay, and RIP assay together demonstrated that miR-342-3p was a target of circVAPA in NSCLC cells. Taken together, we found that circVAPA negatively regulated miR-342-3p expression in NSCLC cells by directly binding to it.

### CircVAPA silencing-mediated anti-tumor effects are largely overturned by the interference of miR-342-3p in NSCLC cells

**Fig. 5:**
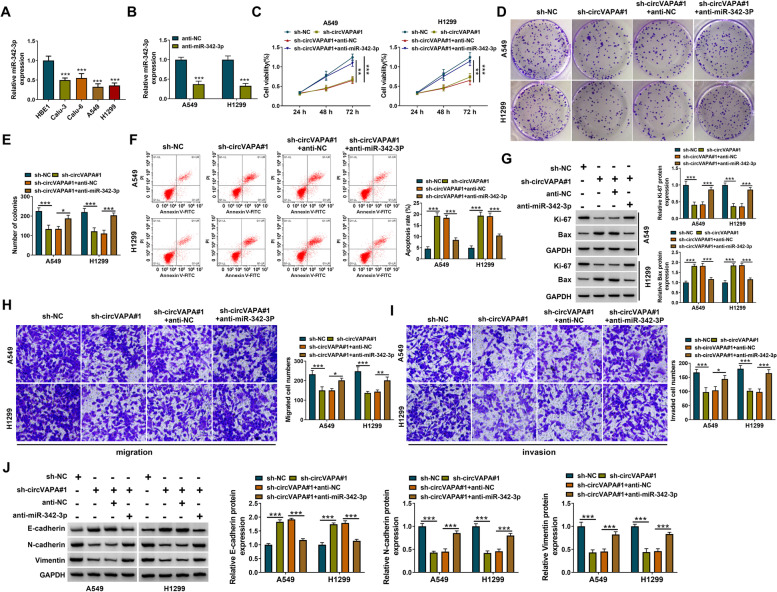
CircVAPA silencing-mediated anti-tumor effects are largely overturned by the interference of miR-342-3p in NSCLC cells. **A** The RT-qPCR assay was performed for examining the expression of miR-342-3p in NSCLC cells and control. **B** The knockdown efficiency of miR-342-3p was confirmed by RT-qPCR assay in A549 and H1299 cells. **C**–**J** A549 and H1299 cells were transfected with si-NC, sh-circVAPA#1, sh-circVAPA#1 + anti-NC, or sh-circVAPA#1 + anti-miR-342-3p. **C**–**E** MTT and colony-forming assays were performed for examining the cell proliferation of A549 and H1299 cells. **F** The apoptotic cells were determined by flow cytometry assay. **G** Western blot assay was conducted to measure the expression of Ki-67 and Bax. (H-I) The transwell assay was conducted to evaluate the migration and invasion of A549 and H1299 cells. **J** The expression levels of E-cadherin, N-cadherin, and Vimentin were evaluated by western blot assay. *P < 0.05, **P < 0.01, ***P < 0.001

The RT-qPCR assay showed that miR-342-3p was downregulated in NSCLC cell lines compared with the HBE1 cell line (Fig. 5A). The knockdown efficiency of anti-miR-342-3p was significant in NSCLC cells (Fig. 5B). CircVAPA knockdown suppressed the proliferation, migration, and invasion and induced the apoptosis of NSCLC cells (Fig. 5C–J). MTT assay and colony-forming assay suggested that the addition of anti-miR-342-3p largely rescued the proliferation of NSCLC cells (Fig. 5C–E). Flow cytometry showed that circVAPA silencing–induced apoptosis of NSCLC cells was largely alleviated by the introduction of anti-miR-342-3p (Fig. 5F). Western blot assay showed that circVAPA knockdown-induced suppressive effect on the expression of Ki-67 and promoting effect on the expression of Bax were both largely reversed by the silence of miR-342-3p (Fig. 5G), suggesting that circVAPA knockdown suppressed the proliferation and induced the apoptosis of NSCLC cells largely by upregulating miR-342-3p. The addition of anti-miR-342-3p also largely recovered the migration and invasion abilities of NSCLC cells (Fig. 5H I). The protein expression of E-cadherin was reduced while the protein levels of N-cadherin and Vimentin were upregulated by the addition of anti-miR-342-3p (Fig. 5J), indicating that circVAPA silencing suppressed the migration and invasion of NSCLC cells largely by elevating miR-342-3p level. These data suggested that circVAPA silencing suppressed the malignant behaviors of NSCLC cells largely by upregulating its target miR-342-3p.

### MiR-342-3p interacts with the 3′UTR of ZEB2 in NSCLC cells

**Fig. 6:**
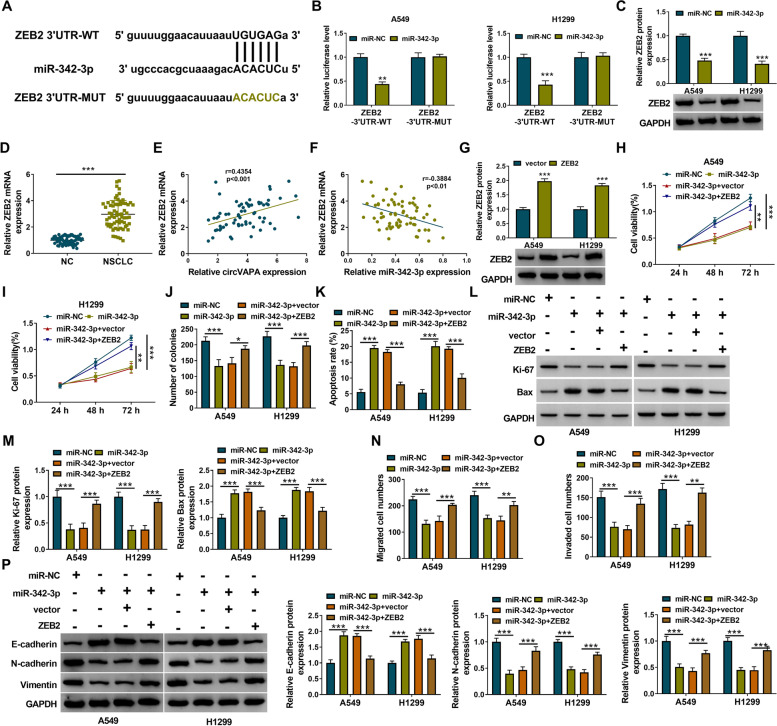
MiR-342-3p interacts with the 3′UTR of ZEB2 in NSCLC cells. **A** Binding regions between miR-342-3p and 3′UTR of ZEB2 were shown. **B** Dual-luciferase reporter assay was performed to show the luciferase activity in A549 and H1299 cells. **C** After transfection with miR-342-3p or miR-NC, the expression of ZEB2 was assessed in A549 and H1299 cells. **D** The expression of ZEB2 was measured by RT-qPCR assay in NSCLC tissues along with adjacent normal tissues. **E**–**F** The correlation relationship between ZEB2 and circVAPA or miR-342-3p was analyzed by Pearson’s correlation analysis. **G** The protein expression of ZEB2 was analyzed by western blot assay in A549 and H1299 cells. **H**–**P** A549 and H1299 cells were transfected with miR-NC, miR-342-3p, miR-342-3p + vector, or miR-342-3p + ZEB2. **H**–**J** The cell proliferation was determined by MTT and colony-forming assays. **K** The flow cytometry assay was conducted to evaluate the apoptosis rate of A549 and H1299 cells. **L**, **M** Western blot assay was conducted to measure the expression of proliferation-associated protein (Ki-67) and pro-apoptotic protein Bax. **N**, **O** The migration and invasion capabilities were examined by transwell analysis. **P** The expression levels of E-cadherin, N-cadherin, and Vimentin were examined by western blot assay. *P < 0.05, **P < 0.01, ***P < 0.001

The putative binding sites between miR-342-3p and the 3′UTR of ZEB2 predicted by the Starbase database are shown in Fig. 6A. The overexpression of miR-342-3p decreased the luciferase activity of the wild-type reporter (ZEB2-3′UTR-WT) but not that of the mutant reporter (ZEB2-3′UTR-MUT) (Fig. 6B), indicating the direct binding relationship between miR-342-3p and the 3′UTR of ZEB2. The overexpression of miR-342-3p reduced the expression of ZEB2 in A549 and H1299 cells (Fig. 6C). ZEB2 mRNA expression was upregulated in NSCLC tissues compared with adjacent normal tissues (Fig. 6D). Furthermore, ZEB2 expression was positively correlated with circVAPA expression while negatively correlated with miR-342-3p expression in NSCLC tissues (Fig. 6E, F). Western blot assay revealed that the overexpression efficiency of the ZEB2 plasmid was significant in NSCLC cells (Fig. 6G). The overexpression of miR-342-3p inhibited proliferation and induced apoptosis of NSCLC cells, which was overturned by overexpression of ZEB2 (Fig. 6H–M). The results of transwell assays suggested that the upregulation of miR-342-3p inhibited the migration and invasion capabilities of NSCLC cells, which was rescued by overexpression of ZEB2 (Fig. 6N, O). In addition, E-cadherin expression was increased, while N-cadherin and Vimentin levels were decreased in miR-342-3p overexpressed-A549 and H1299 cells, which were overturned by transfection with ZEB2 (Fig. 6P). Collectively, miR-342-3p overexpression restrained the malignant phenotypes of NSCLC cells largely by downregulating ZEB2.

### CircVAPA can positively regulate ZEB2 expression by targeting miR-342-3p in NSCLC cells

**Fig. 7:**
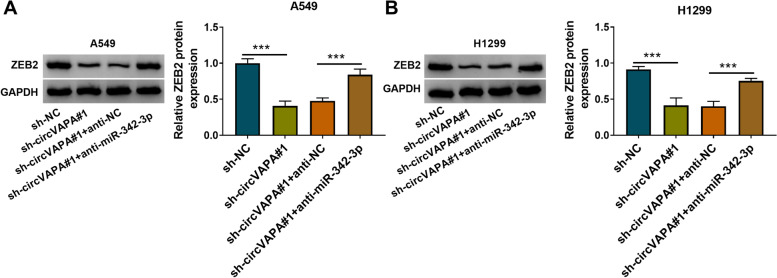
CircVAPA can positively regulate ZEB2 expression by targeting miR-342-3p in NSCLC cells. **A**, **B** The protein expression level of ZEB2 was assessed by western blot assay in A549 and H1299 cells transfected with si-NC, sh-circVAPA#1, sh-circVAPA#1 + anti-NC, or sh-circVAPA#1 + anti-miR-342-3p. ***P < 0.001

The regulatory relationship among circVAPA, miR-342-3p, and ZEB2 was investigated in NSCLC cells. CircVAPA knockdown reduced the protein level of ZEB2, and the addition of anti-miR-342-3p largely recovered the protein expression of ZEB2 in NSCLC cells (Fig. 7A, B). These findings suggested that circVAPA positively regulated the expression of ZEB2 by sponging miR-342-3p.

### CircVAPA silencing suppresses xenograft tumor growth in vivo

**Fig. 8:**
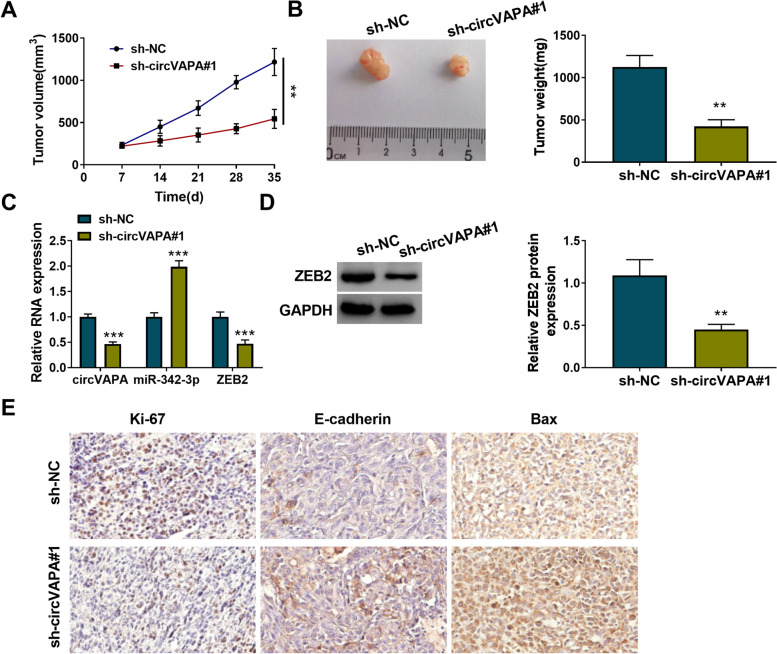
CircVAPA silencing suppresses xenograft tumor growth in vivo. **A** Summary of tumor volume of nude mice was shown. **B** Tumor weight and representative pictures of xenograft in nude mice were presented. **C** The expression levels of circVAPA, miR-342-3p, and ZEB2 were estimated with RT-qPCR assay. **D** Western blot assay was used to measure the ZEB2 level, with GAPDH as control. **E** IHC assay was conducted to analyze the levels of proliferation-associated marker (Ki-67), metastasis-associated marker (E-cadherin), and pro-apoptotic protein (Bax) in xenograft tumor tissues. **P < 0.01, ***P < 0.001

The silence of circVAPA significantly restrained the growth of xenograft tumors (Fig. 8A, B). The expression of the circVAPA/miR-342-3p/ZEB2 axis was determined in xenograft tumor tissues. We found that circVAPA and ZEB2 mRNA expression was downregulated while the miR-342-3p level was upregulated in xenograft tumor tissues in the sh-circVAPA group compared with the sh-NC group (Fig. 8C). In addition, the protein level of ZEB2 was also reduced in the sh-circVAPA group compared with the sh-NC group (Fig. 8D). IHC assay was conducted to determine the staining intensities of proliferation-associated marker (Ki-67), metastasis-associated marker (E-cadherin), and pro-apoptotic protein (Bax) in xenograft tumor tissues to analyze their protein levels. The results revealed that circVAPA knockdown reduced the protein level of Ki-67 and increased the protein levels of Bax and E-cadherin (Fig. 8E), demonstrating that circVAPA silencing restrained the proliferation and metastasis and induced the apoptosis of NSCLC cells in vivo. These findings demonstrated that circVAPA silencing suppressed xenograft tumor growth at least partly by targeting miR-342-3p/ZEB2 axis.

## Discussion

Accumulating evidence have demonstrated that circRNAs can inhibit miRNA function through the competing endogenous mechanism [24, 25]. Previous studies reported that circVAPA is markedly upregulated in multiple human malignancies, and it plays an oncogenic role by regulating cell biological behaviors [7–10]. Consistently, our data suggested that circVAPA was overexpressed in NSCLC tissues and cell lines compared with adjacent normal tissues and normal human bronchial epithelial cell line HBE1. Moreover, NSCLC patients with a high level of circVAPA were associated with poor prognosis. To explore the biological role of circVAPA, loss-of-function experiments were conducted. The results revealed that circVAPA silencing suppressed the proliferation, migration, and invasion and induced the apoptosis of NSCLC cells, suggesting that circVAPA played an oncogenic role in NSCLC progression.

Increasing articles reported that circRNAs can act as miRNA sponges to regulate cell biological behaviors [26, 27]. We found that circVAPA was majorly distributed in the cytoplasmic fraction of NSCLC cells, implying its potential to be a miRNA sponge. In this study, miR-342-3p was identified as the target of circVAPA for the first time. We found that miR-342-3p was downregulated in NSCLC tissues compared with adjacent normal tissues. Previous studies demonstrated that miR-342-3p acted as a tumor suppressor in multiple cancers [28–30], including NSCLC [31, 32]. For instance, Xue et al. demonstrated that miR-342-3p restrains the proliferation and migration abilities of NSCLC cells by targeting AGR2 [32]. Consistent with a former study, we found that miR-342-3p played a tumor suppressor role in NSCLC by suppressing the proliferation, migration, and invasion and inducing the apoptosis of NSCLC cells. Furthermore, circVAPA knockdown-mediated anti-tumor effects were largely overturned by the interference of miR-342-3p, indicating that circVAPA silencing suppressed NSCLC progression largely by upregulating miR-342-3p in vitro.

It is well established that miRNAs can induce the degradation or suppress the translation of target mRNAs by interacting with their 3′UTR [33, 34]. In this study, we found that ZEB2 was a novel target of miR-342-3p for the first time. ZEB2 is reported to be an oncogene in multiple malignancies, including liver cancer [35], breast cancer [36], colorectal cancer [37], esophageal squamous cell carcinoma [38], and NSCLC [39, 40]. For instance, miR-145 is reported to inhibit the motility of NSCLC cells by targeting ZEB2 [39]. miR-132 is reported to restrain the migration and invasion abilities of lung cancer cells by targeting ZEB2 [40]. We found that ZEB2 expression was notably upregulated in NSCLC tissues. In addition, miR-342-3p overexpression-mediated anti-tumor effects in NSCLC cells were largely reversed by the accumulation of ZEB2, suggesting that miR-342-3p restrained NSCLC progression largely by downregulating ZEB2 expression in vitro. CircVAPA can positively regulate the expression of ZEB2 by sponging miR-342-3p in NSCLC cells.

Considering the oncogenic role of circVAPA in vitro, we then explored the role of circVAPA in xenograft tumor growth in vivo. We found that circVAPA knockdown notably restrained the growth of xenograft tumors at least partly through mediating the miR-342-3p/ZEB2 axis.

## Conclusion

In summary, circVAPA was upregulated in NSCLC tissues and cell lines. Mechanistically, circVAPA played an oncogenic role in NSCLC by targeting the miR-342-3p/ZEB2 axis. CircVAPA/miR-342-3p/ZEB2 axis might be a novel target for NSCLC treatment.

## Data Availability

The data sets used and/or analyzed during the current study are available from the corresponding author on reasonable request.
